# Interaction Effect of the Mediterranean Diet and an Obesity Genetic Risk Score on Adiposity and Metabolic Syndrome in Adolescents: The HELENA Study

**DOI:** 10.3390/nu12123841

**Published:** 2020-12-16

**Authors:** Miguel Seral-Cortes, Sergio Sabroso-Lasa, Pilar De Miguel-Etayo, Marcela Gonzalez-Gross, Eva Gesteiro, Cristina Molina-Hidalgo, Stefaan De Henauw, Éva Erhardt, Laura Censi, Yannis Manios, Eva Karaglani, Kurt Widhalm, Anthony Kafatos, Laurent Beghin, Aline Meirhaeghe, Diego Salazar-Tortosa, Jonatan R. Ruiz, Luis A. Moreno, Luis Mariano Esteban, Idoia Labayen

**Affiliations:** 1 Growth, Exercise, NUtrition and Development (GENUD) Research Group, Faculty of Health Sciences, Instituto Agroalimentario de Aragón (IA2), Instituto de Investigación Sanitaria Aragón (IIS Aragón), Universidad de Zaragoza, 50009 Zaragoza, Spain; mseral@unizar.es (M.S.-C.); lmoreno@unizar.es (L.A.M.); 2 Spanish National Cancer Research Centre (CNIO), 28029 Madrid, Spain; ssabroso@cnio.es; 3 CIBER Fisiopatología de la Obesidad y Nutrición (CIBERobn), Instituto de Salud Carlos III, 28029 Madrid, Spain; marcela.gonzalez.gross@upm.es; 4 ImFine Research Group, Department of Health and Human Performance, Facultad de Ciencias de la Actividad Física y del Deporte-INEF, Universidad Politécnica de Madrid, 28040 Madrid, Spain; eva.gesteiro@upm.es; 5 Institute of Nutritional and Food Sciences, Nutritional Physiology, University of Bonn, 53113 Bonn, Germany; 6 EFFECTS 262 Department of Medical Physiology, School of Medicine, University of Granada, 18071 Granada, Spain; cristinamolinapsico@gmail.com; 7 Department of Public Health and Primary Care, Faculty of Medicine and Health Sciences, Ghent University, 9000 Ghent, Belgium; Stefaan.DeHenauw@UGent.be; 8 Department of Pediatrics, Medical School, University of Pécs, 7623 Pécs, Hungary; erhardt.eva@pte.hu; 9 Council for Agricultural Research and Economics-Research Center for Food and Nutrition, 00198 Rome, Italy; laura.censi@crea.gov.it; 10 Department of Nutrition and Dietetics, School of Health Science & Education, Harokopio University, 176 71 Athens, Greece; manios@hua.gr (Y.M); ekaragl@hua.gr (E.K.); 11 Division of Gastroenterology and Hepatology, Department of Internal Med III, Austria and Austrian Academic Institute for Clinical Nutrition, 1090 Vienna, Austria; kurt.widhalm@meduniwien.ac.at; 12 Faculty of Medicine, University of Crete, 715 00 Crete, Greece; kafatos@med.uoc.gr; 13 CIC-1403-Inserm-CHU, Clinical Investigation Center, LIRIC UMR 995 Inserm, CHU Lille, Université de Lille, 59000 Lille, France; Laurent.BEGHIN@CHRU-LILLE.FR; 14 UMR1167, RID-AGE, Risk Factors and Molecular Determinants of Aging-Related Diseases, Centre Hosp, Institut Pasteur de Lille, Université de Lille, 59019 Lille, France; aline.meirhaeghe@pasteur-lille.fr; 15 Department of Ecology and Evolutionary Biology, University of Arizona, Tucson, AZ 85719, USA; dftortosa@email.arizona.edu; 16 PROmoting FITness and Health through Physical Activity Research Group (PROFITH), Department of Physical and Sports Education, School of Sports Science, Sport and Health University Research Institute (iMUDS), University of Granada, 18016 Granada, Spain; ruizj@ugr.es; 17 Escuela Politécnica de La Almunia, Universidad de Zaragoza, 50100 Zaragoza, Spain; lmeste@unizar.es; 18 Department of Health Sciences, Public University of Navarra, 31006 Pamplona, Spain; idoia.labayen@unavarra.es

**Keywords:** metabolic syndrome, Mediterranean diet, genetic risk score, HELENA, adolescents, sex

## Abstract

Obesity and metabolic syndrome (MetS) are worldwide major health challenges. The Mediterranean diet (MD) is associated with a better cardiometabolic profile, but these beneficial effects may be influenced by genetic variations, modulating the predisposition to obesity or MetS. The aim was to assess whether interaction effects occur between an obesity genetic risk score (obesity-GRS) and the MD on adiposity and MetS in European adolescents. Multiple linear regression models were used to assess the interaction effects of an obesity-GRS and the MD on adiposity and MetS and its components. Interaction effects between the MD on adiposity and MetS were observed in both sex groups (*p* < 0.05). However, those interaction effects were only expressed in a certain number of adolescents, when a limited number of risk alleles were present. Regarding adiposity, a total of 51.1% males and 98.7% females had lower body mass index (BMI) as a result of higher MD adherence. Concerning MetS, only 9.9% of males with higher MD adherence had lower MetS scores. However, the same effect was observed in 95.2% of females. In conclusion, obesity-related genotypes could modulate the relationship between MD adherence and adiposity and MetS in European adolescents; the interaction effect was higher in females than in males.

## 1. Introduction

Metabolic syndrome (MetS) is known to be a major world health challenge, with increasing prevalence together with obesity and cardiovascular diseases [1]. The prevalence of overweight and obesity worldwide has drastically increased among youth in recent years, with similar numbers in males and females [2]. The prevalence of obesity and metabolic syndrome in European children and adolescents continue in the same increasing line despite the efforts of prevention programs in recent years [3,4]. The definition of MetS features a number of cardiometabolic risk factors, including total and/or central adiposity, dyslipidemia, hypertension, and insulin resistance [5]. Clustering of cardiometabolic risk factors is increasingly considered in children’s and adolescents’ health rather than single risk factors [6,7]. In European children, an inverse association between the Mediterranean diet (MD) and childhood obesity has been observed [8] and showed that high MD adherence at early age is associated with a lower risk of developing overweight and obesity during childhood [9]. Moreover, in children and adolescents, MD was associated with lower body mass index (BMI) and improved glucose and lipid profiles [10]. The beneficial effects associated with a high MD adherence may be influenced by the interaction with other factors, such as genetic variations, which could modulate the predisposition/risk to obesity and MetS [11]. In adults, a systematic review [12] showed that the interaction between the melanocortin 4 receptor (*MC4R*) gene (a protein-coding gene previously associated to BMI [13]) and MD modulates the development of obesity and type 2 diabetes mellitus (T2DM) phenotypes. In Chinese children and adolescents, interactions between genetic variants and dietary behaviors in relation to obesity have been observed [14].

Previous studies have shown the potential of genetic approaches that identify individuals at high risk of developing a disease. That is the case of genetic risk scores (GRS), that combine a number of single nucleotide polymorphisms (SNPs) by summing the number of risk alleles [15]. In order to try to prevent the development of obesity and MetS in European adolescents it is crucial to improve our understanding of the predisposing genetic factors [16,17].

To our knowledge, no studies have examined the interaction effect between MD adherence and an obesity-related GRS on adiposity and MetS in European adolescents. Therefore, the aim of our study is to assess whether interaction effects occur between the MD adherence and obesity-GRS on adiposity and MetS in European adolescents. We hypothesize that higher predisposition to obesity risk may attenuate the protective effect of MD adherence on adiposity and MetS in European adolescents.

## 2. Materials and Methods

### 2.1. Study Design and Population

**Figure 1:**
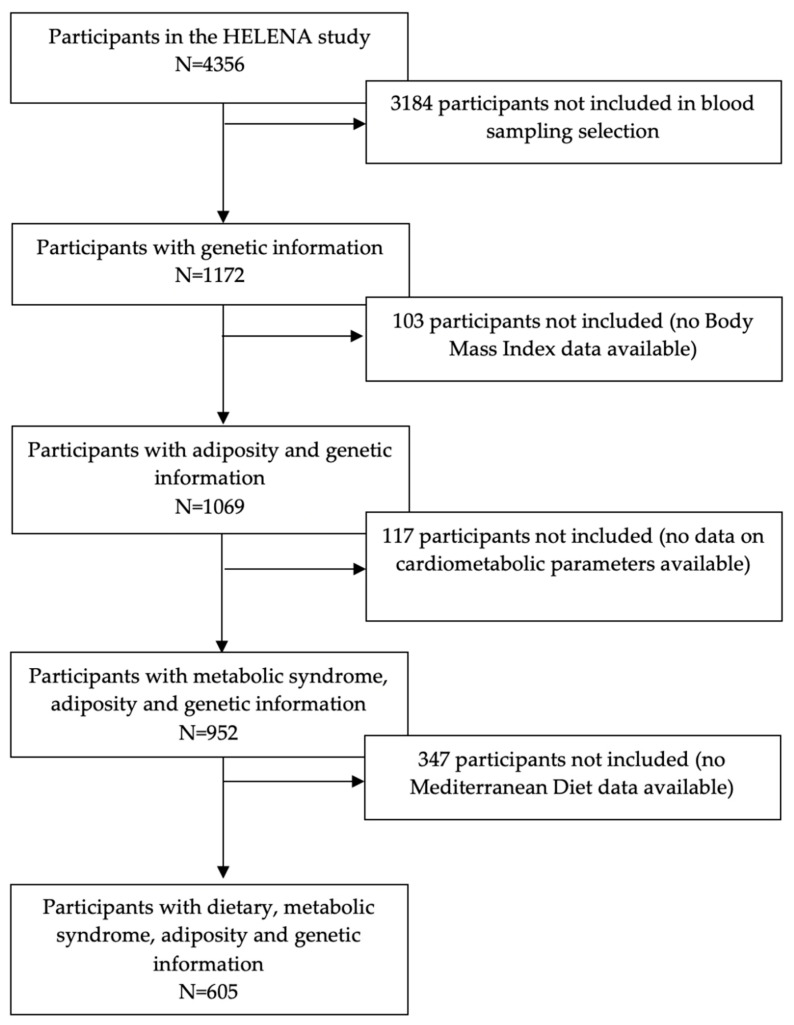
Flow chart of the sample selection process.

The Healthy Lifestyle in Europe by Nutrition in Adolescence (HELENA) multicentric and cross-sectional study included a total sample of 4356 adolescents (51.6% females), aged 11–19 years [18]. Data were obtained from 10 European cities located in different geographical points within Europe, during 2006–2007. The HELENA study was designed to obtain reliable and comparable data on nutrition and health-related parameters, applying standardized procedures [19]. The HELENA study was approved by the Research Ethics Committees of each study site and followed the ethical guidelines of the Declaration of Helsinki 1964 (revision of 2000), good clinical practice, and the legislation about clinical research in humans in each one of the countries involved in the study [20]. Written informed consent was obtained by the parents/legal guardians of all participants. Blood sampling was performed in one third of the individuals randomly selected from the total sample (N = 1172) [19]. Inclusion of specific parameters to develop the present study (genomic, adiposity, cardiometabolic risk factors, and dietary data) provided a final number of 605 adolescents (51.6% female) meeting the selection criteria. Information of selection procedure is displayed in a flow chart (Figure 1).

### 2.2. Physical Examination, Adiposity Measurements and Cardiometabolic Risk Score

Anthropometric measurements were performed by trained researchers following standard protocols [21]. Height was measured barefoot in the Frankfort plane with a telescopic height measuring instrument (Type SECA 225) to the nearest 0.1 cm, and weight was measured in underwear and without shoes with an electronic scale (Type SECA 861) to the nearest 0.1 kg. BMI was calculated from weight and height (kg/m^2^) [22]. Waist circumference (WC) was measured in triplicate with a nonelastic tape (SECA 200) to the nearest 0.1 cm as the mid-point between the lowest rib and the iliac crest [21], and the average of the three measures was used. Systolic blood pressure (SBP) and diastolic blood pressure (DBP) were measured twice in sitting position separated in a 10- minute interval with a blood pressure oscillometric monitor device OMRON HEALTHCARE^®^ (M6-HEM7001; OMRON HEALTHCARE^®^, Kyoto, Japan) The lowest blood pressure (BP) reading was used. Thereafter, the mean of arterial pressures (MAP) of all participants was obtained from the DBP + [(SBP − DBP)/3)] formula. Total cholesterol (TC), HDL-cholesterol (HDL-c), triglycerides (TG), and glucose were measured using enzymatic methods (Dade Behring, Schwalbach, Germany). Insulin levels were obtained from frozen serum using an Immulite 2000 analyzer (DPC Bierman GmbH, Bad Nauheim, Germany). As measurement of insulin resistance, the homeostatic model assessment (HOMA) index was calculated from glucose and insulin measurements [23]. Moreover, a clustered cardiometabolic risk score was computed from the sum of the standardized z-scores of TC/HDL ratio, WC, HOMA index, and MAP [7]. The standardized z-scores of intended variables were calculated from the age and gender specific cut-off points [24]. Lower values in the score indicate better cardiometabolic risk profile. As sensitivity analyses, a second MetS risk score, comprising HDL-c, WC, Glucose, SBP and TG, was obtained from the International Diabetes Federation (IDF) guidelines [5]. HDL-c was multiplied by −1 as characterized by lower metabolic risk with increasing values. Results obtained with both cardiometabolic risk scores in adolescents have been included in the present analyses.

### 2.3. Dietary Intake Assessment and Mediterranean Diet Score

Dietary habits were determined from a self-administered, computerized, validated 24-h dietary recall called the HELENA Dietary Assessment Tool (HELENA-DIAT) [25,26], a tool validated first in Flemish adolescents [26] and then adapted to be used in the 10 cities [27]. Participants completed the HELENA-DIAT on two non-consecutive days within a time span of two weeks. This method has been used and recommended to assess dietary intake in European children and adolescents [28]. In order to calculate individual usual dietary intake, the multiple source method (MSM) was used [28]. This method allows correction of dietary data for between and within individuals’ variability.

We used a Mediterranean diet score (MDS) based on nine single components: vegetables, fruits and nuts, cereals and roots, pulses, fish, monounsaturated/saturated fatty acids ratio, dairy products, meat, and alcohol. A scale indicating the degree of adhesion/adherence to the traditional MD was developed [29]. The description of MD food subgroup components is described elsewhere [30]. Vegetables, fruits and nuts, cereals, legumes, fish, dairy products, and unsaturated to saturated fat ratio positively contributed to the MD adherence, whereas meat (including processed meat) and alcohol consumption were inversely considered. Of note is that dairy products are positively considered as they are recommended during growth and development periods, such as adolescence [31]. Alcohol intake was regarded as an unhealthy habit among adolescents. Therefore, in a no-alcohol consumption situation, the value 1 was assigned, while any alcohol intake was computed as value 0. The MD adherence was constructed by a 0 to 9 points scale, with higher scores indicating greater adherence [32]. The sex-specific median intake (g/day) of all subgroups forming the MDS is shown in Supplementary Table S1.

### 2.4. Genomic Information and Genetic Risk Score

Standard methods for blood collection, transport, and analysis was performed by a certified laboratory [33]. Blood sampling (EDTA K3 tubes) for DNA extraction, collection, and storage was performed at the Institute of Nutritional and Food Sciences (IEL) of the University of Bonn, and sent to the Laboratoire d’Analyse Genomique Centre de Ressources Biologiques (LAG-CRB) BB- 0033-00071 Institut Pasteur de Lille, F-59000 Lille, France. DNA was obtained from white blood cells with the Puregene kit (QIAGEN, Courtaboeuf, France) and stored at −20 °C. The genotyping was done by an Illumina system (Illumina, Inc., San Diego, CA, USA) using the Golden Gate technology (sampling procedure scheme, GoldenGate; Software, Inc, San Francisco, CA, USA).

To analyze the influence of genetic information on the association between MD and adiposity and cardiometabolic biomarkers, we used the obesity-GRS developed from HELENA adolescents (submitted but not yet accepted) [34] using 21 SNPs significantly associated with overweight/obesity, defined as the equivalent to BMI > 25kg/m^2^. The main characteristics of SNPs forming the obesity-GRS are displayed in Supplementary Table S2. Also, a comparative analysis by sex is shown in Supplementary Table S3.

### 2.5. Statistical Analysis

To test the variables´ normality, the Shapiro–Wilk test was performed. As not all variables follow a normal distribution, the descriptive sex-specific characteristics are displayed as median and interquartile range (IQR) for continuous variables; absolute and relative frequencies are shown for categorical variables. In order to compare differences by sex, the statistical Pearson’s chi-square test was used for categorical variables and the Mann–Whitney–Wilcoxon test for continuous variables.

Sex-specific multiple linear regression models were used to assess the association between adiposity and cardiometabolic parameters and the interaction effect between the obesity-GRS and MD, adding to the effect of MD alone in the same model.

RStudio Version 1.2.5001 (RStudio Team (2015). RStudio: Integrated Development for R. RStudio, Inc., Boston, MA, USA, URL http://www.rstudio.com/) was used to perform all statistical analyses and *p* < 0.05 was the significance level set in the present analysis.

## 3. Results

### 3.1. Descriptive Characteristics of the Study Sample

**Figure 2:**
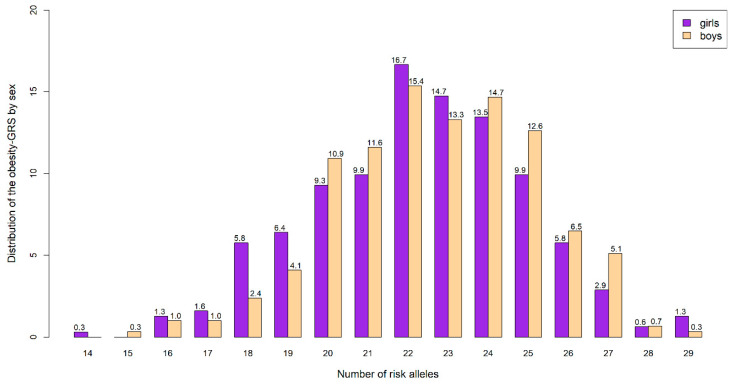
Distribution of the obesity-GRS (obesity-related genetic risk score) by sex (% displayed) in the HELENA cohort according to the number of risk alleles.

The main characteristics of participants are shown in Table 1. In summary, there were significant differences between males and females for weight and height (*p* ≤ 0.001) although no significant differences were found for BMI. Regarding cardiometabolic risk factors, girls had higher WC (*p* ≤ 0.001) and HOMA (*p* = 0.044), whereas boys showed higher SBP (*p* ≤ 0.001), MAP (*p* ≤ 0.001), and MetS (*p* ≤ 0.001) than girls. There were no significant differences for the remaining cardiometabolic parameters. The distribution of obesity-GRS among participants is displayed by sex in Figure 2. Focusing in the SNPs included in the obesity-GRS, no statistically significant differences between sex were found in Supplementary Table S3.

### 3.2. Interaction between MD and Obesity-GRS on Adiposity/Cardiometabolic Variables

Table 2 shows the association between the cardiometabolic parameters in relation to MD and the interaction between MD and the obesity-GRS in the additive model, by sex groups.

Considered within the additive model (Table 2), MD adherence had a protective role over BMI (male *p* < 0.01 vs. female *p* ≤ 0.001), WC (male *p* < 0.05 vs. female *p* ≤ 0.001), and MetS (male *p* < 0.05 vs. female *p* ≤ 0.01) in both sex groups. However, the association of the MD with HOMA (*p* < 0.05) and SBP (*p* < 0.05) was only observed in females, and the association of the MD with MAP (*p* ≤ 0.05) and DBP (*p* < 0.05) only in males.

Furthermore, both the MD and the interaction between MD and obesity-GRS were significant in the case of BMI for both sex groups; the inverse association between the MD and BMI was higher in females than in males (females *p* ≤ 0.001 vs. males *p* < 0.01). When studying the cardiometabolic variables, we observed significant interactions between obesity-GRS and MD in both sex groups for WC (*p* < 0.05) and MetS (*p* < 0.05). For both adiposity and cardiometabolic variables, females showed stronger interactions than males. Moreover, the obesity-GRS and MD showed a significant interaction on MAP (*p* < 0.05) and DBP (*p* < 0.01) for males only, whereas the interaction on HOMA was only significant for females (*p* < 0.05).

**Figure 3:**
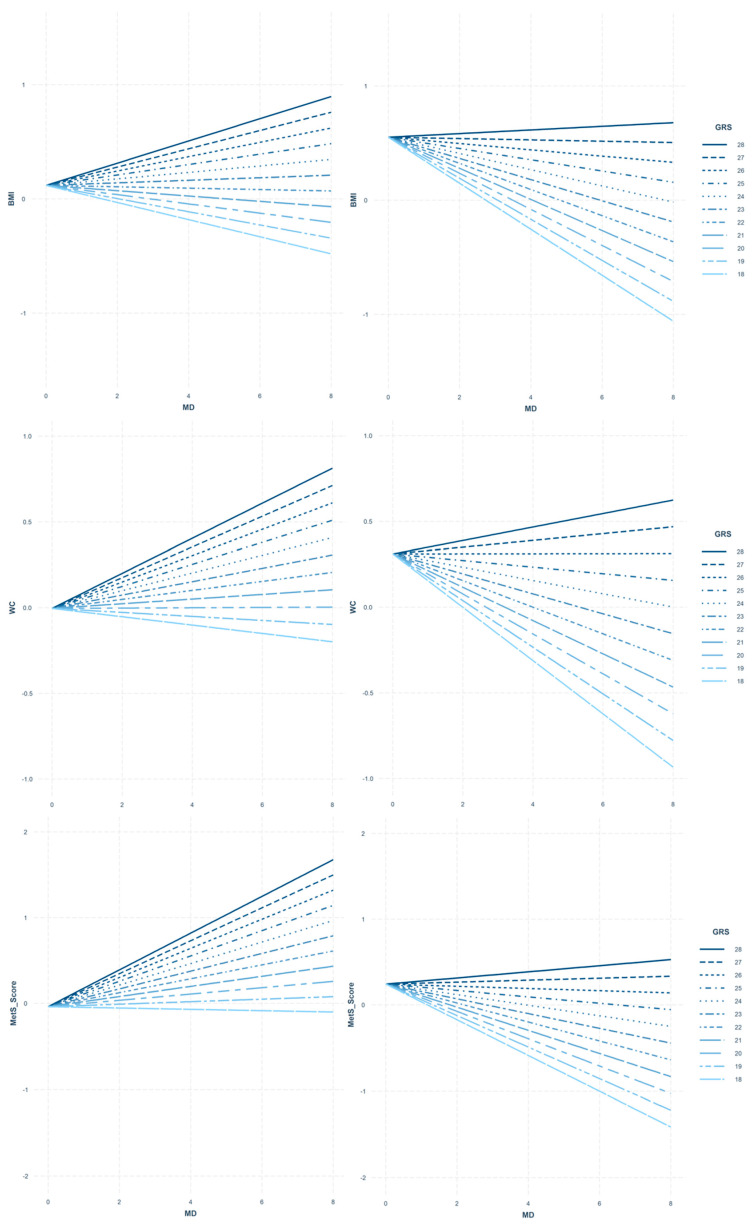
Matrix panel of interaction models between body mass index (BMI), waist circumference (WC), and metabolic syndrome score (MetS Score); and the Mediterranean diet (MD) according to the obesity genetic risk score (Obesity-GRS) modulation compared by sex (males left panel, females right panel). Obesity-GRS values (18–28) displayed according to our population distribution. Legend: when designing the population distribution representation, different lines were drawn as reference points to observe the trend of the studied population according to the genetic predisposition to obesity. When analyzing the results represented in these figures, a positive gradient shows the MD acting as risk factor, whereas a negative gradient indicates the protective role of the MD.

Figure 3 shows the interaction effects of the obesity-GRS and the MD on BMI, WC, and MetS for male and female participants. The relations between the MD and BMI, WC, and MetS were modulated by the obesity-GRS values. In order to interpret the abovementioned variables, different sub-figures have been displayed in a matrix panel according to each sex group for BMI, WC, and MetS. Different lines were drawn to relate MD and adiposity and cardiovascular biomarkers modulated by the distribution of the genetic predisposition to obesity in our population. It must be remarked that the majority of the adolescents were concentrated in the central parts of the graph, within 20-26 risk alleles (82.3% of the total population); therefore, extreme values should be interpreted cautiously. Thus, for those participants represented with a negative slope, a higher MD adherence could act as a protective factor in relation to cardiometabolic factors despite their genetic predisposition to obesity (high or low).

Regarding the adiposity parameters, a total of 46.8% of males (those with 22 risk alleles or below) with higher MD adherence had lower BMI; in 98.1% of females (in those individuals scoring 27 risk alleles or below), a higher MD adherence was associated with lower BMI, attenuating the genetic risk to obesity. For WC, we also observed that 19.8% males (≤20 risk alleles) with higher MD adherence, had lower WC; however, 95.2% of females (≤26 risk alleles), had also lower WC levels.

Concerning MetS, 4.8% of males with higher MD adherence had lower cardiometabolic risk score if the risk score was ≤18. More so, 95.2% of females having higher MD adherence showed higher MetS scores if the risk score was ≤26. In terms of sensitivity analyses, the MetS analyses were repeated using the MetS score following the IDF recommendations. In males, the obesity-GRS and MD showed a significant interaction on MetS (*p* = 0.001) but not on the IDF MetS score (*p* = 0.092). However, the interaction was significant for both MetS scores in females (*p* = 0.005 vs. *p* = 0.003).

There were sex-related differences in the interaction between genes and diet on other cardiometabolic parameters, such as HOMA, DBP, and MAP. Specifically, in females with higher MD adherence, those having ≤25 risk alleles had lower HOMA levels (89.4% of the adolescents). Male participants with higher MD adherence with an obesity-GRS ≤21 (31.4% of the adolescents) had lower DBP. Likewise, males with higher MD adherence and an obesity-GRS ≤22 (46.8% of the adolescents) had a lower MAP. The interaction effects of the obesity-GRS and MD on HOMA, DBP, and MAP for male and female participants are displayed in Supplementary Figure S1.

## 4. Discussion

The main findings of the present study indicate that the influence of high MD adherence on adiposity and MetS was only expressed when a limited number of risk alleles were present. As a result, the gene–diet interaction effect was higher in females than in males.

The MD has previously been shown to provide numerous health benefits [35], such as the reduction of risk factors for non-communicable diseases [36,37]. However, little is known about how the genetic variations among individuals determine the response to MD adherence [38]. To our knowledge, no previous studies have assessed their interaction effects using an obesity-related GRS. Instead, isolated SNPs from candidate genes previously associated in the literature with adiposity or MetS were examined. More so, no gene–diet interaction studies on adiposity and cardiometabolic parameters considering the MD were found in adolescents, as the majority of studies have been conducted in adult populations.

Concerning adiposity, similar findings were observed in a study where the *FTO*rs9939609 and *MC4R*rs17782313 polymorphisms showed an interaction with the MD adherence, which reduced the risk of obesity and T2DM [39]. In line with our findings, one study also showed low adiposity levels in relation with the interaction effect with different allele combinations, and considering other dietary approaches different to the MD: low polyunsaturated fatty acids (PUFA) intake showed an inverse association with obesity risk (BMI ≥ 30 kg/m^2^) when the *ADAM*17i33708A polymorphism was present [40]. Another study, considering high saturated fat intake, showed a significantly higher BMI in the GG carriers of the *THRA*rs1568400 than in the A carriers [41], suggesting counter-productive effects in comparison to our study. In the current study, not only BMI, but also WC, a surrogate marker of abdominal adiposity, showed lower levels in both male and female adolescents, as a result of the obesity-GRS–MD interaction; to our knowledge, no similar findings have been reported in other studies with similar characteristics.

Regarding MetS, we found no studies assessing a gene–diet interaction effect on a MetS risk score. We have also assessed cardiometabolic parameters individually. Our results showed low HOMA levels in the majority of females modulated by the obesity-GRS when adhering to the MD. One study assessing the effect of increasing the ratio of saturated fat to carbohydrate intake, showed higher HOMA levels in carriers of the minor allele (*PLIN*11482G>A) [42].

Concerning DBP, the male adolescents with higher MD adherence had lower levels of DBP when having 21 or fewer risk alleles. Our findings are coincident to one intervention study promoting the Dietary Approaches to Stop Hypertension (DASH) diet, where AA carriers of the Angiotensinogen genotype (G-6A ANG polymorphism) showed the greatest reduction in DBP [43]. No interaction studies considering the MAP levels were reported in the literature.

Despite the fact that we did not observe an effect on HDL-c, we included the TC:HDL ratio variable in our MetS score, showing a significant association with the gene–diet interaction. In this sense, Ordovás et al. observed that women carriers of the A allele of the *APOA1* gene (G-A polymorphism) responded with higher HDL-c concentrations to a high PUFA intake, whereas the opposite effect was observed in the G carriers [44]. Other authors have also pointed novel genes–nutrients interactions with high-carbohydrate diets in GG carriers of *KCTD10*i5642G>C and *MMAB*3U3527G>C and C allele carriers of *KCTD10*V206VT>C, contributing to lower HDL-c concentrations [45].

When adhering to the MD, no associations were found in terms of TG levels. However, another study showed that, after 12 months of a MD-based intervention, higher levels of HDL-c and TG were seen in those individuals carrying the T allele of the *CETP*rs3764261 than in those with the GG genotype [46]. Another study showed that the *TNF-alpha*rs1800629 GG subjects had higher levels of TG than A carriers in MetS patients after another MD intervention [47]. Finally, other authors assessed the interaction between *PPARA*L162V and PUFA intake, noticing lower TG levels with higher intake in the V carriers [48].

In order to compare the present results to other cardiometabolic definitions, the development of our MetS score was compared to another MetS score, following the IDF recommendations. The IDF score showed no association with the GRS–MD interaction in males. This fact could be due to the different age and gender specific criteria selected to define the cut-off points between authors. Nevertheless, positive associations of the GRS–MD interaction were seen in the female group for both MetS scores. The consensus to use unified criteria to identify adolescents at risk of MetS remains under development.

The present study has some limitations. As the HELENA project is a cross-sectional study, cause–effect relationships cannot be established. Moreover, only selected risk loci are available in the HELENA study. When constructing the obesity-GRS, it has been calculated that BMI changes can be explained by a small proportion of genetic variants discovered so far [49], so potential rarer variants yet to be found might emerge when Genome-Wide Association Studies (GWAS) are carried out.

On the other hand, the study presents several strengths. Most analyses from previous studies were focused on specific single SNPs interactions, whereas the present study used an obesity-GRS, considered as a useful genetic tool [50], to predict adiposity and MetS in European adolescents. More so, the development of the cardiometabolic risk score was considered appropriate for the present study as it provides higher sensitivity and low susceptibility to errors compared to other approaches [51]. Furthermore, we included the whole MD pattern developing a cluster of different food groups in an adherence scale rather than considering single macronutrients or individual specific food groups´ intake. Additionally, different effects were identified in males and females. There is different behavior dependent on sex, not attributable to genetic predisposition, maybe associated with physical activity. Due to the multicentric design of the HELENA study, congregating participants from 10 European cities, the researchers have been provided with large datasets from diversely distributed populations across Europe. Finally, the study is focused on adolescents, a population age that is understudied and where early detection plays a key role in the development of obesity and metabolic syndrome. Little has been found in the literature using a similar approach, where most similar studies are available on adult populations.

As MetS and excess of adiposity may occur at any stage from childhood to adulthood, early detection and diagnosis is fundamental to elaborate on health prevention programs among youth to effectively reduce the risk of cardiovascular diseases and T2DM [52,53]. At the same time, it has been previously shown that greater adherence to MD was associated with a significant improvement in overall health status among youth, suggesting the implementation of a MD dietary pattern for primary prevention of major chronic diseases [54]. The effect of the interventions would be heterogeneous depending on the genetic background of the individuals and should be considered in the efficacy analyses.

## 5. Conclusions

Obesity-related genotypes had a modulation effect in the relationship between MD adherence and obesity and MetS risk in European adolescents. The genes–diet interaction effect on MetS was stronger in females than in males. These observations strengthen the idea of applying genomic information to promote targeted dietary advice.

## Figures and Tables

**Table 1:** Demographics and cardiometabolic characteristics of the Healthy Lifestyle in Europe by Nutrition in Adolescence (HELENA) participants displayed by sex.

	Total	Male	Female	*p*-Value
	*n* = 605	*n* =293	*n* =312	
Age (years)	14.7 (13.8–15.6)	14.8 (13.8–15.6)	14.8 (13.7–15.7)	0.948
Height (cm)	166.0 (159.5–172.2)	170.0 (163.9–177.0)	162.2 (157.0-167.2)	**<0.001**
Weight (kg)	58.4 (49.9–64.5)	61.1 (51.1–68.8)	55.8 (49.2–61.6)	**<0.001**
BMI (kg/m^2^)	21.1 (18.6–22.9)	21.0 (18.5–22.7)	21.2 (18.7–23.0)	0.194
WC (cm)	72.1 (66.7–75.8)	65.75 (46.0–79.0)	71.5 48.7–83)	**<0.001**
HOMA index	2.2 (1.3–2.6)	2.2 (1.3–2.5)	2.3 (1.4–2.7)	**0.044**
SBP (mmHg)	116 (108–124)	120 (112–129)	112 (105–120)	**<0.001**
DBP (mmHg)	64.4 (59.0–70.0)	64.0 (59.0-69.0)	64.8 (60.0–70.0)	0.331
MAP	0.6 (−0.01–1.1)	1.1 (0.5–1.6)	0.2 (−0.4–0.7)	**<0.001**
HDL-c (mmol/L)	55.7 (49–63)	53.3 (47-59)	57.9 (50–65)	**<0.001**
TG (mmol/L)	68.7 (47.0–80.0)	65.7 (46.0–79.0)	71.5 (48.7–83.0)	0.056
TC:HDL ratio	2.3 (2.5–1.1)	2.3 (2.5–3.2)	2.9 (2.5–3.3)	0.839
PA (mins/day)	54.8 (40.7–71.5)	65.4 (51.8–82.4)	47.3 (35.2–59.8)	**<0.001**
MetS Score *	0.02 (-1.2–1.0)	0.3 (−0.7–1.3)	−0.3 (−1.5–0.8)	**<0.001**
MDS **	4 (0–8)	4 (0–8)	4 (0–8)	0.495
Obesity-GRS ***	23 (21–24)	23 (21–25)	22 (21–24)	0.087

Abbreviations: BMI (body mass index); WC (waist circumference); HOMA index (homeostatic model assessment index); SPB (systolic blood pressure); DBP (diastolic blood pressure); MAP (mean arterial pressure); HDL-c (high density lipoprotein cholesterol); TG (triglycerides); TC:HDL ratio (total cholesterol/HDL cholesterol ratio); PA (physical activity); (MetS Score (Metabolic Syndrome score); MDS (Mediterranean diet score); and obesity-GRS (obesity-related genetic risk score). Median values (p. 25–p. 75) expressed. * Metabolic Syndrome score resulting from the mean of WC, HOMA, MAP, and TC-HDL variables combined. ** Mediterranean diet score resulting from the sum of nine food subgroups compliance. *** Genetic risk score resulting from the sum of risk alleles of HELENA participants. Boldface values indicate sig *p*-value Sig *p*-value < 0.05.

**Table 2:** Multiple linear regression models of obesity-related genetic risk score (obesity-GRS) and Mediterranean diet (MD) interaction, and the MD effect alone on adiposity and cardiometabolic parameters by sex.

*p*-Values	Male		Female	
	Obesity-GRSxMD	MD	Obesity-GRSxMD	MD
BMI (kg/m^2^)	**0.003**	**0.008**	**<0.001**	**<0.001**
WC (cm)	**0.009**	**0.030**	**<0.001**	**<0.001**
HOMA	0.495	0.836	**0.027**	**0.013**
SBP (mmHg)	0.994	0.739	0.310	**0.047**
DBP (mmHg)	**0.005**	**0.014**	0.795	0.626
MAP	**0.031**	**0.045**	0.872	0.325
TG (mmol/L)	0.421	0.413	0.587	0.689
TC:HDL	0.465	0.530	0.184	0.118
MetS Score	**0.014**	**0.047**	**0.006**	**0.002**

Boldface values indicate sig *p*-value Sig *p*-value < 0.05.

## Supplementary Materials

The following are available online at https://www.mdpi.com/2072-6643/12/12/3841/s1, Table S1: Mediterranean diet score items specified in g/day displayed in sex-specific median intake in HELENA participants with dietary information available, Table S2: Main characteristics of the 21 single nucleotide polymorphisms (SNPs) included in the obesity genetic risk score (obesity-GRS), Table S3: Comparative analysis of the 21 single nucleotide polymorphisms (SNPs) included in the obesity genetic risk score (obesity-GRS) by sex, Figure S1: Interaction models between HOMA, diastolic blood pressure (DBP) and mean arterial pressure (MAP) and Mediterranean Diet (MD) according to the obesity genetic risk score (obesity-GRS) modulation in female. Obesity-GRS values (18–28) are displayed according to our population distribution.
