# Supplementary material for: Interaction Effect of the Mediterranean Diet and an Obesity Genetic Risk Score on Adiposity and Metabolic Syndrome in Adolescents: The HELENA Study
Source: Nutrients. 2020 Dec 16;12(12):3841. doi: 10.3390/nu12123841 (PMC7766705; doi:10.3390/nu12123841)
Supplement: Supplementary file 1 [file nutrients-12-03841-s001.pdf]

## SUPPLEMENTAL MATERIAL

**Table 1.** Mediterranean Diet Score items specified in g/day displayed in sex–specific median intake in HELENA participants with dietary information available.

| | **Male** | **Female** | |
|---|---|---|---|
| | **n=293** | **n=312** | ***p*–Value** |
| Vegetables (g/day) | 82.50 (50.62–130.45) | 87.43 (52.51–124.06) | 0.773 |
| Fruits and nuts (g/day) | 95.73 (44.16–177.92) | 112.03 (45.48–179.73) | 0.648 |
| Cereal roots (g/day) | 316.98 (265.39–414.40) | 262.01 (205.93–303.98) | ≤0.001 |
| Pulses (g/day) | 1.90 (0.87–3.52) | 1.38 (0.68–3.39) | 0.036 |
| Fish (g/day) | 12.14 (0–29.30) | 12.69 (5.99–25.91) | 0.901 |
| Dairy products (g/day) | 210.08 (95.16–366.19) | 148.72 (65.28–260.00) | ≤0.001 |
| Meat (g/day) | 157.38 (112.26–207.29) | 121.04 (77.77–165.84) | ≤0.001 |
| Alcohol (g/day) | 0.53 (0.21–0.98) | 0.27 (0.02–0.46) | ≤0.001 |
| FU/FS ratio | 0.85 (0.77–0.99) | 0.86 (0.77–0.98) | 0.989 |
| MDS | 4 (0–8) | 4 (0–8) | 0.495 |
| High adherence (N, %) | 128 (43.7%) | 139 (56.3%) | 0.894 |

Median values (p25–p75) displayed. FU/FS ratio: unsaturated to saturated fatty acids ratio. Boldface values indicate sig p–value Sig p–value <0.05.

**Table 2.** Main characteristics of the 21 single nucleotide polymorphisms (SNPs) included in the obesity genetic risk score (Obesity–GRS).

| rs code | Nearest Gene | Alleles (Major/Minor) | MAF | *p* | Genotyping success rate | HWE |
|---|---|---|---|---|---|---|
| rs2010899 | *AMPD1* | A/C | 0.44 | 0.012 | 99.8 | 0.349 |
| rs4135275 | *PPARG* | A/G | 0.19 | 0.024 | 99.9 | 0.655 |
| rs4912905 | *NR3C1* | C/G | 0.23 | 0.004 | 100.0 | 0.665 |
| rs7701443 | *NR3C1* | A/G | 0.40 | <0.001 | 100.0 | 0.127 |
| rs13182800 | *NR3C1* | C/A | 0.24 | <0.001 | 99.9 | 0.183 |
| rs9355296 | *LPA* | G/A | 0.13 | 0.013 | 100.0 | 0.216 |
| rs1524107 | *IL–6* | G/A | 0.07 | 0.006 | 100.0 | 0.879 |
| rs3211867 | *CD36* | C/A | 0.07 | 0.033 | 100.0 | 0.728 |
| rs2183013 | *CNTFR* | C/G | 0.17 | 0.001 | 100.0 | 0.934 |
| rs2515362 | *CNTF* | A/G | 0.44 | 0.024 | 99.9 | 0.184 |
| rs1800497 | *DRD2* | G/A | 0.18 | 0.049 | 99.8 | 0.763 |
| rs1019731 | *IGF1* | C/A | 0.11 | 0.005 | 100.0 | 0.744 |
| rs9939609 | *FTO* | T/A | 0.40 | <0.001 | 100.0 | 0.322 |
| rs4783961 | *CETP* | A/G | 0.50 | 0.014 | 100.0 | 0.558 |
| rs8068149 | *NOS2A* | G/A | 0.46 | 0.010 | 100.0 | 0.136 |
| rs7502966 | *THRA* | A/G | 0.44 | 0.025 | 99.7 | 0.264 |
| rs1568400 | *THRA* | A/G | 0.26 | 0.023 | 100.0 | 0.349 |
| rs4246444 | *FASN* | C/A | 0.27 | 0.008 | 94.7 | 0.461 |
| rs1044250 | *ANGPTL4* | G/A | 0.29 | 0.005 | 99.6 | 0.051 |
| rs17373080 | *LXRβ* | G/C | 0.32 | 0.005 | 99.7 | 0.523 |
| rs2143511 | *PTPN1* | A/G | 0.43 | 0.004 | 99.9 | 0.337 |

**Table S3.** Comparative analysis of the 21 single nucleotide polymorphisms (SNPs) included in the obesity genetic risk score (Obesity–GRS) by sex.

| | Allele distribution (0, 1, 2) | | |
|---|---|---|---|
| **rs code** | **Male** | **Female** | *p* |
| rs2010899 | 94 (32.1), 132 (45.1), 67 (22.9) | 82 (26.3), 169 (54.2), 61 (19.6) | 0.079 |
| rs4135275 | 198 (67.6), 85 (29.0), 10 (3.4) | 198 (63.5), 105 (33.7), 9 (2.9) | 0.458 |
| rs4912905 | 157 (53.6), 116 (39.6), 20 (6.8) | 176 (56.4), 113 (36.2), 23 (7.4) | 0.692 |
| rs7701443 | 95 (32.5), 150 (51.2), 48 (16.4) | 118 (37.8), 151 (48.4), 43 (13.8) | 0.338 |
| rs13182800 | 165 (56.3), 105 (35.8), 23 (7.8) | 193 (61.9), 101 (32.4), 18 (5.8) | 0.319 |
| rs9355296 | 232 (79.2), 58 (19.8), 3 (1.0) | 223 (71.5), 85 (27.2), 4 (1.3) | 0.089 |
| rs1524107 | 258 (88.1), 34 (11.6), 1 (0.3) | 278 (89.1), 33 (10.6), 1 (0.3) | 0.921 |
| rs3211867 | 260 (88.7), 31 (10.6), 2 (0.7) | 264 (84.6), 47 (15.1), 1 (0.3) | 0.217 |
| rs2183013 | 213 (72.7), 70 (23.9), 10 (3.4) | 214 (68.6), 89 (28.5), 9 (2.9) | 0.421 |
| rs2515362 | 101 (34.5), 139 (47.3), 53 (18.1) | 92 (29.5), 151 (48.4), 69 (22.1) | 0.298 |
| rs1800497 | 191 (65.2), 95 (32.4), 7 (2.4) | 222 (71.2), 79 (25.3), 11 (3.5) | 0.129 |
| rs1019731 | 229 (78.2), 62 (21.2), 2 (0.7) | 246 (78.8), 63 (20.2), 3 (1.0) | 0.402 |
| rs9939609 | 117 (39.9), 132 (45.1), 44 (15.0) | 113 (36.2), 145 (46.5), 54 (17.3) | 0.575 |
| rs4783961 | 71 (24.2), 147 (50.2), 75 (25.6) | 82 (26.3), 155 (49.7), 75 (24.0) | 0.816 |
| rs8068149 | 91 (31.1), 135 (46.1), 67 (22.9) | 87 (27.9), 148 (47.4), 77 (24.7) | 0.675 |
| rs7502966 | 91 (31.1), 140 (47.8), 62 (21.2) | 113 (36.2), 148 (47.4), 51 (16.3) | 0.215 |
| rs1568400 | 160 (54.6), 116 (39.6), 17 (5.8) | 161 (51.6), 124 (39.7), 27 (8.7) | 0.377 |
| rs4246444 | 159 (54.3), 116 (39.6), 18 (6.1) | 177 (56.7), 109 (39.4), 26 (8.3) | 0.360 |
| rs1044250 | 149 (50.9), 109 (37.2), 35 (11.9) | 173 (55.4), 107 (34.3), 32 (10.3) | 0.510 |
| rs17373080 | 133 (45.4), 132 (45.1), 28 (9.6) | 146 (46.8), 133 (42.6), 33 (10.6) | 0.809 |
| rs2143511 | 96 (32.8), 136 (46.4), 61 (20.8) | 103 (33.0), 145 (46.5), 64 (20.5) | 0.662 |

Legend: Allele distribution according to number of risk alleles: 0=no risk alleles; 1=one risk allele and 2=two risk alleles. Relative frequency (%) values shown in brackets.

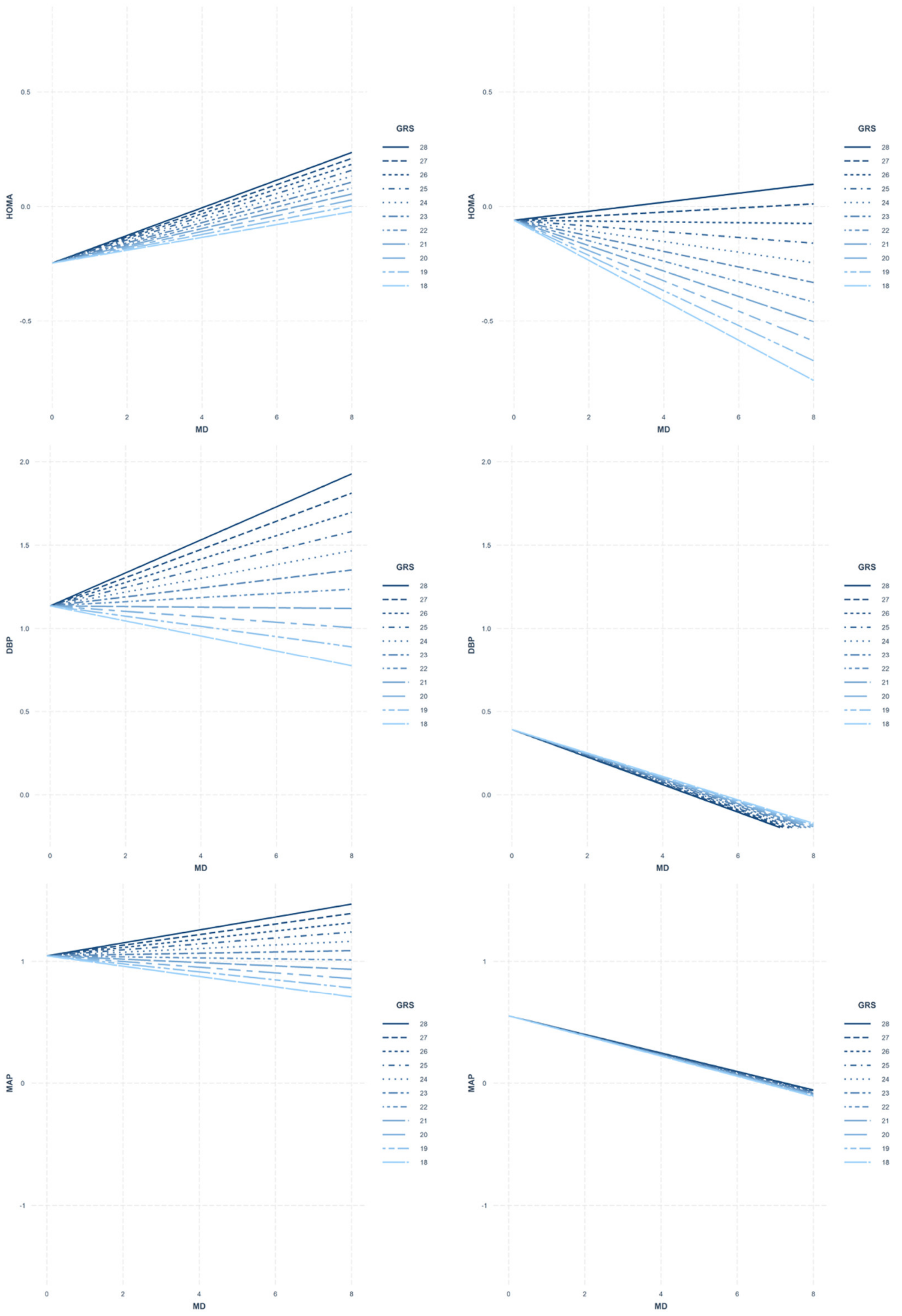


**Figure 1.** Interaction models between HOMA, diastolic blood pressure (DBP) and mean arterial pressure (MAP) and Mediterranean Diet (MD) according to the Obesity Genetic Risk Score (Obesity–GRS) modulation in box sex. Obesity–GRS values (18–28) displayed according to our population distribution (males left panel, females right panel). Obesity–GRS values (18–28) displayed according to our population distribution. Legend: When designing the population distribution representation, different lines were drawn as reference points to observe the trend of the studied population according to the genetic predisposition to obesity. When analyzing the results represented in these figures, a positive gradient shows the MD acting as risk factor whereas a negative gradient indicates the protective role of MD.
